# Sulopenem Disk Development, Quality Control Range, and MIC to Disk Result Correlation for *Enterobacterales* Isolates

**DOI:** 10.1128/jcm.00246-23

**Published:** 2023-06-26

**Authors:** Sailaja Puttagunta, Steven I. Aronin, Jeanna DiFranco-Fisher, Laura Koeth

**Affiliations:** a Iterum Therapeutics, Old Saybrook, Connecticut, USA; b Laboratory Specialists, Inc., Westlake, Ohio, USA; Johns Hopkins University

**Keywords:** antibacterial agent, antimicrobial susceptibility testing, disk diffusion, sulopenem, susceptibility

## Abstract

Sulopenem disk masses of 2, 5, 10, and 20 μg were evaluated by susceptibility testing isolates by broth microdilution and disk diffusion. A 2-μg disk was chosen, and error-rate bounding analysis in accordance with Clinical and Laboratory Standards Institute (CLSI) guideline M23 was conducted using a proposed sulopenem susceptible/intermediate/resistant (S/I/R) interpretive criterion of ≤0.5/1/≥2 μg/mL. Among the evaluated *Enterobacterales* (*n* = 2,856), very few interpretive errors were observed (no very major errors and only one major error). An eight-laboratory quality control (QC) study was performed using the 2-μg disk, and 99.0% (470/475) of results were within a 7-mm range of 24 to 30 mm. Results were similar by disk lot and media, and no outlier sites were observed. A sulopenem 2-μg disk QC range for Escherichia coli 29522 of 24 to 30 mm was established by the CLSI. A 2-μg sulopenem disk performs accurately and reproducibly for testing of *Enterobacterales*.

## INTRODUCTION

Sulopenem is a thiopenem β-lactam antibiotic with intravenous and oral formulations being developed for the treatment of infections caused by multidrug-resistant bacteria. Sulopenem etzadroxil, the oral prodrug of intravenous sulopenem, is combined with probenecid to extend its plasma half-life (1). As seen with other β-lactams, sulopenem’s bactericidal mode of action against susceptible bacteria is due to binding to cell-wall penicillin binding proteins to block peptidoglycan cross-linking and prevent further cell wall synthesis. Sulopenem possesses potent activity against species of the *Enterobacterales* that encode extended-spectrum β-lactamases (ESBLs) or AmpC-type (AmpC) β-lactamases that confer resistance to third-generation cephalosporins. The targeted Gram-negative spectrum of sulopenem is balanced by its *in vitro* activity against anaerobic pathogens, which is similar to that of imipenem. Sulopenem is affected by resistance mechanisms that have been well-characterized for other carbapenem-class agents. For *Enterobacterales*, where resistance is typically mediated by carbapenemases, cross-resistance between sulopenem and comparator carbapenems is expected; among lactose-nonfermenting Gram-negative bacilli (e.g., Pseudomonas aeruginosa and Acinetobacter baumannii), where multidrug resistance is more common, carbapenem resistance mediated by carbapenemases, porin loss, or efflux pump expression affects the activity of sulopenem in similar fashion to that observed with comparator carbapenems. Regardless of pre-existing resistance to carbapenems, sulopenem has no appreciable activity against P. aeruginosa. Sulopenem is in phase 3 clinical development and is not yet approved for clinical use.

Pathogens commonly associated with urinary tract and intrabdominal infections are becoming increasingly resistant to antibiotics used to treat these infections, and new oral/IV agents are needed to address this issue (2). Previous *in vitro* studies demonstrated that the activity of sulopenem was unaffected by nonsusceptibility to trimethoprim-sulfamethoxazole and/or ciprofloxacin, extended-spectrum β-lactamases, or AmpC (1).

Laboratory testing of isolates to determine antibiotic susceptibility is an important component of ensuring appropriate treatment to prevent development of antibiotic-resistant isolates. While not used as widely as automated testing devices, disk diffusion testing is a good option for laboratory testing of antimicrobial agents. Among the first antimicrobial susceptibility testing devices to be approved for a new antibiotic is the disk for disk diffusion testing. For example, the plazomicin disk was approved 15 days post FDA approval of plazomicin for treatment of complicated urinary tract infections (cUTI) (see https://www.fda.gov/drugs and https://www.fda.gov/medical-devices/510k-clearances/search-releasable-510k-database). Disks are one of the few manual devices available during the phase between FDA approval of the antibiotic and approval of automated testing systems (3). Disk diffusion testing has many advantages including low cost, the ability to select and test multiple antibiotics on a single agar plate, and the lack of a requirement for special microbiology laboratory equipment (4). When disks for new antibiotics are developed, the development of the disk is typically conducted using standardized methodology according to the CLSI or the European Committee on Antimicrobial Susceptibility Testing (EUCAST). Recently, however, the two organizations harmonized their approach to disk development, and new disks are developed using joint CLSI and EUCAST recommendations for disk content selection (5).

Prior to the availability of CLSI M23S (5), initial work regarding determining disk content for sulopenem disk diffusion susceptibility testing was conducted in 2006. Sulopenem disk masses of 5, 10, and 20 μg were evaluated by testing target pathogens for susceptibility by broth microdilution and disk diffusion. A subsequent study in 2018 evaluated 2-μg and 5-μg sulopenem disk masses and the 2-μg disk was ultimately chosen for additional studies and for use in the phase 3 clinical trials. The sulopenem disk development program, including disk potency studies and the establishment of quality control (QC) results for the sulopenem disk for *Enterobacterales* isolates, are further described herein.

## MATERIALS AND METHODS

### Disk evaluation studies. (i) Sulopenem 5-, 10-, and 20-μg disk development.

Sulopenem powder was provided by Pfizer Inc. (New York City, NY, USA) and 5-, 10-, and 20-μg disks were prepared by the study vendor (The Clinical Microbiology Institute [CMI], Wilsonville, OR, USA). Broth microdilution was performed according to CLSI document M7-A7, 2006 (6), and disk diffusion was performed according to the CLSI document M2-A9, 2006 (7) using the same lot of sulopenem powder and in-house prepared disks. MIC trays were produced at CMI using cation-adjusted Mueller-Hinton broth (CAMHB) (Difco; Becton, Dickinson, Sparks, MD, USA). Comparator antibiotics included ertapenem and imipenem (data not shown). Concurrent inocula of *Enterobacterales* isolates (*n* = 116; *Enterobacter* spp., *n* = 12; Escherichia coli, *n* = 15; ESBL-positive [ESBL^+^] E. coli, *n* = 11; Klebsiella pneumoniae, *n* = 15; ESBL^+^ K. pneumoniae, *n* = 12; Morganella morganii, *n* = 12; *Proteus* spp., *n* = 14; *Providencia* spp., *n* = 6; Serratia marcescens, *n* = 6; *Salmonella* spp., *n* = 6; *Shigella* spp., *n* = 7; see the supplemental material) were tested by broth microdilution and disk diffusion and included quality control (QC) strains such as E. coli ATCC 25922. Carbapenem-resistant isolates were not available for testing in this study. When a value was outside of the normally established QC ranges for comparator agents and initial expected QC ranges for sulopenem, the test was repeated the following workday. In every instance, the repeat testing fell within the CLSI established QC ranges. At the time of the study, quality control ranges for sulopenem had not been established; however, all sulopenem MIC QC results were within the range of 0.015 to 0.06 μg/mL (the current CLSI sulopenem MIC QC range for E. coli ATCC 25922) Correlation of the results obtained by the CLSI reference broth microdilution method (BMD) and diffusion method were calculated.

### (ii) Sulopenem 2- and 5-μg disk development.

The 2-μg and 5-μg sulopenem disks were prepared by the manufacturer Liofilchem (Roseto degli Abruzzi, Italy). The same lot of sulopenem powder was used for both the manufacture of the disk and the preparation of broth microdilution plates. Broth microdilution and disk diffusion testing of the test disk were conducted using CLSI methodology (8, 9) using both wild-type and resistant clinical *Enterobacterales* isolates (*n* = 43; E. coli, *n* = 8; ESBL^+^ E. coli, *n* = 15; K. pneumoniae, *n* = 1; ESBL^+^ K. pneumoniae, *n* = 1; carbapenem-resistant *Enterobacterales*, *n* = 16; Enterobacter cloacae, *n* = 2; see the supplemental material) with a range of sulopenem MIC values. Correlation of the results obtained by the CLSI reference broth microdilution method (BMD) and diffusion method were calculated. Colony counts were determined for at least 10% of clinical isolates (include at least one of each species) for both the broth microdilution and disk diffusion testing.

The reproducibility of the disk was assessed using the ATCC quality control strains including E. coli ATCC 25922. E. coli ATCC 25922 was tested on three separate days in triplicate with a different inoculum prepared for each test for two lots of Mueller-Hinton agar (MHA) from two different manufacturers (BD-BBL and Oxoid). In addition, reproducibility was assessed by testing five quality control strains at least 20 times and calculating a mean zone diameter for each strain.

### (iii) Sulopenem clinical trial data: MIC and 2-μg disk testing.

International Health Management Associates (Schaumburg, IL, USA) served as the central laboratory for the clinical trial and performed reidentification, susceptibility testing, and genotypic testing of clinical trial isolates, including PCR and whole-genome sequencing of phenotypically resistant isolates. Broth microdilution was performed according to CLSI methodology (9) for concurrent inocula of *Enterobacterales* isolates (*n* = 2,856; *Citrobacter* spp., *n* = 64; *Enterobacter* spp., *n* = 81; E. coli, *n* = 2,148 [includes 355 ESBL^+^ E. coli isolates]; K. pneumoniae, *n* = 302 [includes 87 ESBL^+^ K. pneumoniae isolates]; *Klebsiella* spp., *n* = 83; M. morganii, *n* = 30; Proteus mirabilis, *n* = 89; *Proteus* spp., *n* = 11; *Providencia* spp., *n* = 8; S. marcescens, *n* = 14; *Serratia* spp., *n* = 2; *Salmonella* spp., *n* = 1; other *Enterobacterales* spp., *n* = 23; see the supplemental materials). Scatterplots were generated for isolates from the uncomplicated urinary tract infections (uUTI), cUTI, and IAI clinical trials, as well as the Liofilchem 2-μg disk study.

### MIC disk analysis.

In accordance with CLSI M23, the error-rate bonded method was used for analysis, and categorical error rates (very major error [VME], major error [ME], and minor error [mE]) were determined based on all results and the following MIC result subsets (error rate acceptance rates): ≥ intermediate (I) + 2 dilutions (<2% VME and <5% ME), I ± 1 dilution (<10% VME and ME and <40% mE), and ±I − 2 dilutions (<2% mE). For the assessment based on all results, the acceptable discrepancy rates were <1.5% VME and <3% ME (10).

### Establishment of quality control ranges for Escherichia coli ATCC 25922.

Two lots of sulopenem 2-μg disks were used in this study, both manufactured by Liofilchem (Roseto degli Abruzzi, Italy). The comparator disk was a single-lot meropenem disk manufactured by Becton, Dickinson (Sparks, MD, USA), and all results for the comparator were as expected (data not shown). Three lots of Mueller-Hinton agar media from three different manufacturers, (BBL [Becton, Dickinson], Sparks, MD, USA), Hardy (Santa Maria, CA, USA), and Remel (Lenexa, KS, USA), were used. Eight laboratories participated in the study, and each site tested 10 replicates over a 3-day period according to the CLSI disk diffusion method (11). Zones were measured to the nearest whole millimeter using a sliding caliper or ruler and read at complete inhibition (within the inside zone). The results were analyzed using both Rangefinder and the Gavin statistic (4).

## RESULTS

### Broth microdilution to disk correlation.

In the initial disk development study of the 5-, 10-, and 20-μg disks of 116 *Enterobacterales*, zone sizes exceeded 30 mm for a large proportion of the disk results. For the 20-μg disk, the zone sizes ranged from 12 to 41 mm with 84% of results ≥30 mm. For the 10-μg disk, the zone sizes ranged from 8 to 36 mm, with 50% of results ≥30 mm. For the 5-μg disk, the zone sizes ranged from 6 to 33 mm, with 18% of zone diameters ≥30 mm. E. coli ATCC 25922 BMD MIC results were within the current CLSI range of 0.015 to 0.016 μg/mL.

**FIG 1:**
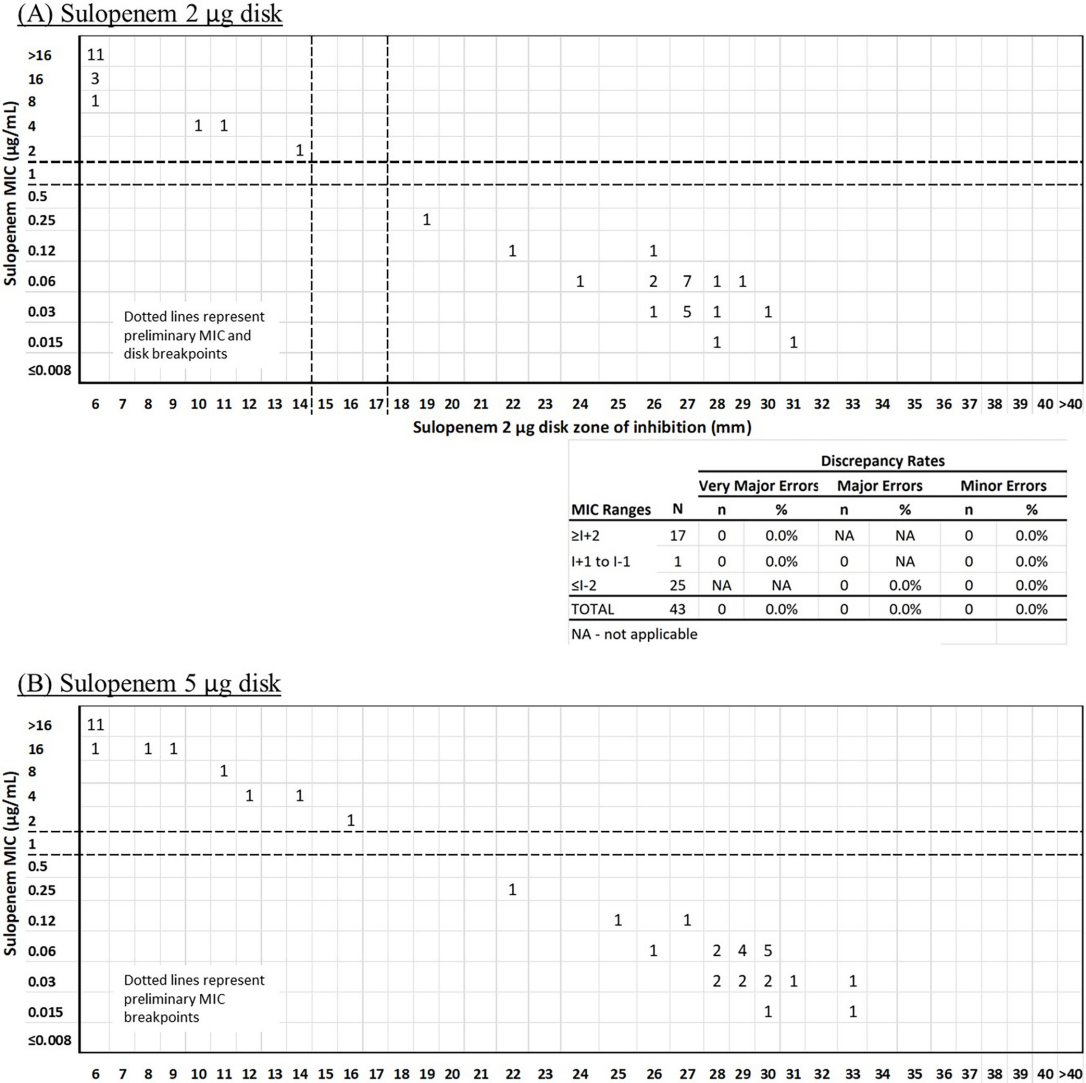
Sulopenem 2- and 5-μg disk development. Correlation between sulopenem broth MIC values (μg/mL) and disk diffusion zone diameters (mm) for *Enterobacterales* (*n* = 43). (A) Sulopenem 2-μg disk. (B) Sulopenem 5-μg disk.

In the subsequent 2- and 5-μg study of 43 *Enterobacterales*, the 5-μg disk zone sizes also ranged from 6 to 33 mm with 11 zone diameters of ≥30 mm, and the 2-μg disk zone sizes ranged from 6 to 31 mm with only two results with zone diameters of ≥30 mm (Fig. 1). The 2020 EUCAST-CLSI document recommendation is that zone diameters for wild-type organisms should ideally fall between 15 and 35 mm (ideally not above 30 mm) (5). The choice of the 2-μg disk best met these zone size requirements. In addition, the 2-μg disk demonstrated smaller zone sizes for *Enterobacterales* isolates with known carbapenemases (range, 6 to 14 mm; there was at least a 6-mm difference between disk diameter zones for wild-type isolates compared to carbapenemase-producing isolates) (see the supplemental material). The 2-μg disk was ultimately chosen for additional studies and for use in the phase 3 clinical trials. The 2-μg disk produced highly reproducible zone diameters with all QC strain results falling within ±1 mm for all strains (refer to “Sulopenem 2- and 5-μg study quality control strain reproducibility”).

### Clinical trial results.

**FIG 2:**
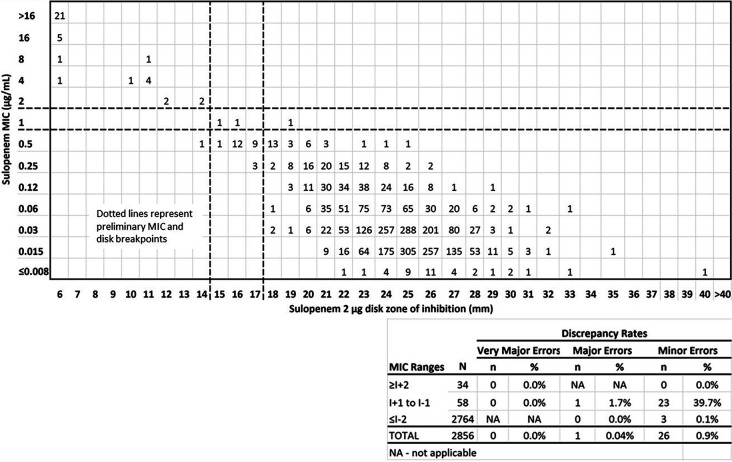
Sulopenem 2-μg disk clinical trial results. Correlation between sulopenem broth MIC values (μg/mL) and disk diffusion zone diameters (mm) for *Enterobacterales* (*n* = 2,856).

**FIG 3:**
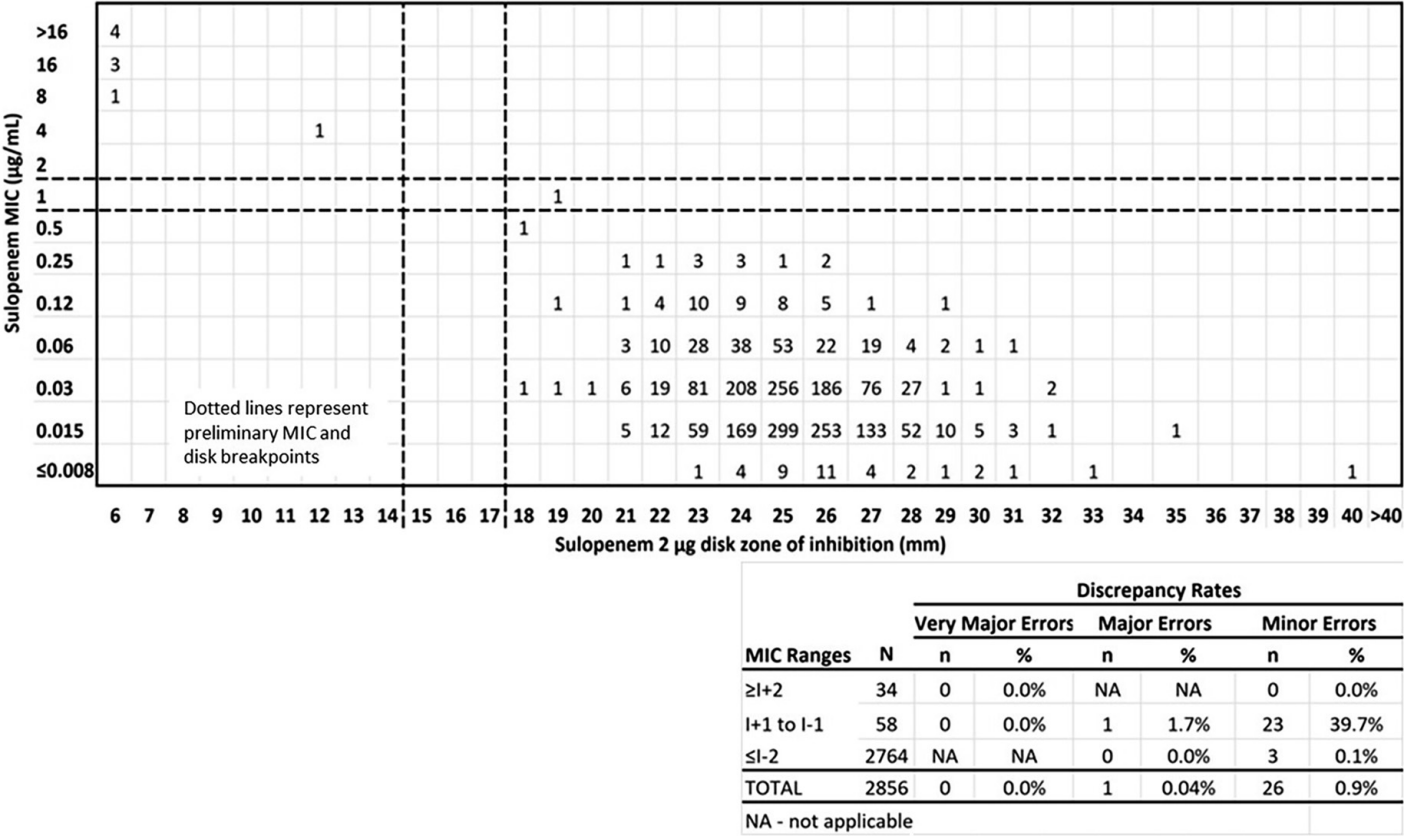
Sulopenem 2-μg disk clinical trial results. Correlation between sulopenem broth MIC values (μg/mL) and disk diffusion zone diameters (mm) for *Enterobacterales* E. coli (*n* = 2,148).

Error-rate bounding analysis in accordance with CLSI guideline M23 is presented in Fig. 2. The scatterplot was generated for isolates using MIC and disk data from the sulopenem Phase 3 IAI (IT001-303 cIAI trial (AKA SURE-3), NCT03358576), cUTI (IT001-302 cUTI trial (AKA SURE-2), NCT03357614), and uUTI (IT001-301 uUTI trial (AKA SURE-1), NCT03354598) clinical trials (https://clinicaltrials.gov) and the Liofilchem 2- and 5-μg disk development study using proposed sulopenem broth interpretive criteria of ≤0.5/1/≥2 μg/mL (susceptible/intermediate/resistant). As shown by scatterplot, using disk interpretive criteria of ≥18 mm susceptible, 15 to 17 mm intermediate, and ≤14 mm resistant results in very few interpretive errors with no very major errors and only one major error among the evaluated *Enterobacterales* (*n* = 2,856). For E. coli, only one minor error was observed using these proposed breakpoints, exceeding the CLSI M23 (10) thresholds slightly where few isolates were available for analysis (Fig. 3). There was very good separation of the 2-μg sulopenem disk results based on MIC results categorized as distinctly susceptible and resistant (excluding results within ±1 dilution of the intermediate range). Disk results of ≤11 mm correlate exclusively to MIC results of 4 to 16 μg/mL, and disk results of ≥17 mm correlate exclusively to MIC results of ≤0.008 to 0.25 μg/mL (with 96.5% of disk results in the 21- to 28-mm range).

The CLSI established MIC and disk ranges for E. coli ATCC 25922 were available at the time of clinical trial testing, and all sulopenem MIC values generated during clinical trial testing for quality control strain, E. coli ATCC 25922, were within the CLSI established range. All but one sulopenem zone diameter was within the CLSI established range for E. coli ATCC 25922, and in that instance, the result was 1 mm below the accepted range; results were in QC 5 days prior to that instance and 8 days after at the central microbiology lab.

### Sulopenem 2- and 5-μg study quality control strain reproducibility.

E. coli ATCC 25911 mean zones (mm) for initial triplicate testing of the 2-μg sulopenem disk on four lots of MHA were as follows: BBL lot 1, 27; BBL lot 2, 26; Oxoid lot 1, 28; and Oxoid lot 2, 26. Quality control strains were tested 20 times with the 2-μg sulopenem disk. The disk was reproducible with all results falling within ±1 mm for all tested strains on testing days. The range and mean measurements of the results are presented in Table 1.

**Table 1:** Quality control results

Quality control strain ATCC number	Range of 2-μg sulopenem disk zones (mm)	Mean of 2-μg sulopenem disk zones (mm)	Inoculum CFU/mL
E. coli 25922	26–30^*a*^	28	1.3 × 10^8^
E. faecalis 29212	6–8	7	1.0 × 10^8^
S. aureus 29213	31–34	32	1.2 × 10^8^
S. pneumoniae 49619	23–32	29	0.9 × 10^8^
H. influenzae 49766	25–28	27	0.8 × 10^8^

a CLSI expected range, which was established after this study, is 24 to 30 mm.

### Eight laboratory results for establishment of E. coli ATCC 29522 quality control range for the sulopenem 2-μg disk.

**FIG 4:**
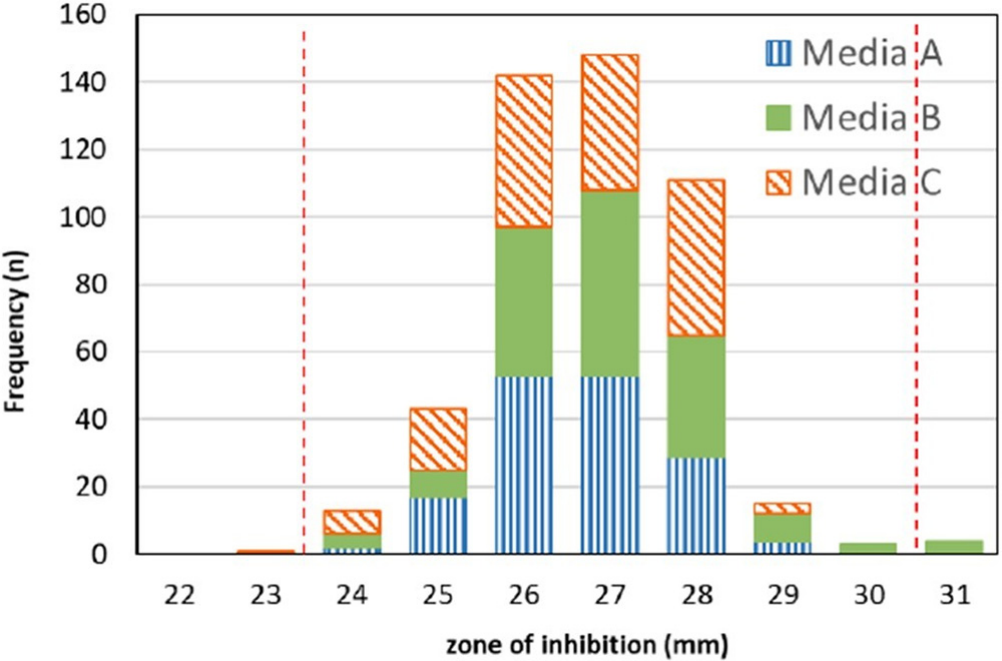
E. coli ATCC 25922 sulopenem 2-μg disk eight-laboratory quality control results. Distribution of disk zone diameters (mm) by media lot.

**FIG 5:**
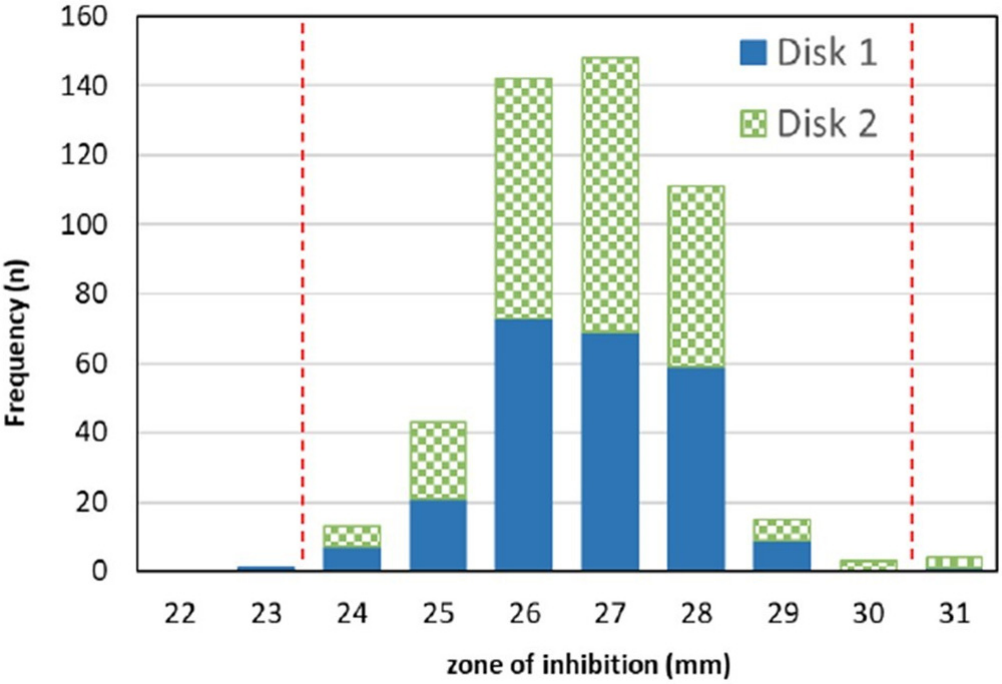
E. coli ATCC 25922 sulopenem 2-μg disk eight-laboratory quality control results. Distribution of disk zone diameters (mm) by disk lot.

For the sulopenem 2-μg disk, a total of 99.0% (470/475) of results were within a 7-mm range of 24 to 30 mm (Table 2). In addition, results were similar by disk lot (mean zones were 26.5 and 26.8 mm) and media (mean zones were 26.3 to 27.0 mm) (Fig. 4 and 5). Minimal variability was observed between sites for the sulopenem disk (mean zones were between 25.7 and 27.8 mm). The average colony count was 1.2 × 10^8^ CFU/mL (range of 4.1 × 10^7^ to 1.9 × 10^8^ CFU/mL). For the meropenem 10-μg disk, a total of 98.3% of meropenem results were within the established QC range, with a mean zone size of 30.3 mm (data not shown). There are no outlier sites, according to the Rangefinder analysis of the sulopenem 2-μg disk results. Therefore, the CLSI approved QC range for the sulopenem 2-μg disk was determined to be 24 to 30 mm (12).

**Table 2:** Distribution of sulopenem 2-μg disk zone diameters by disk, media, and testing sites for E. coli ATCC 25922^*a*^

Zone (mm)	Distribution													
	Disk 1	Disk 2	Media A	Media B	Media C	Lab 1	Lab 2	Lab 3	Lab 4	Lab 5	Lab 6	Lab 7	Lab 8	All labs
**22**														**0**
**23**	1				1				1					**1**
**24**	7	6	2	4	7		2		9			2		**13**
**25**	21	22	17	8	18	8	8		8	1		17	1	**43**
**26**	73	69	53	44	45	40	12	13	34	10	1	29	3	**142**
**27**	69	79	53	55	40	11	28	28	6	18	12	11	34	**148**
**28**	59	52	29	36	46	1	8	18		22	46	1	15	**111**
**29**	9	6	4	8	3		2	1	1	7	1		3	**15**
**30**		3	1	2					1	2				**3**
**31**	1	3	1	3									4	**4**
**Total**	240	240	160	160	160	60	60	60	60	60	60	60	60	480
**Mean**	26.5	26.8	26.3	27.0	26.7	26.1	26.6	27.1	25.7	27.5	27.8	25.9	27.5	**26.8**
**Median**	26	27	26	27	27	26	27	27	26	28	28	26	27	**27**
**Mode**	26	27	26	27	28	26	27	27	26	28	28	26	27	**27**
**Minimum**	23	24	24	24	23	25	24	26	23	25	26	24	25	**23**
**Maximum**	31	31	31	31	29	28	29	29	30	30	29	28	31	**31**
**No. in range**	9	8	8	8	7	4	6	4	8	6	4	5	7	**9**

a Rangefinder, 99.0% within 7-mm range of 24 to 30 mm; Gavan statistic, 99.0% within 7-mm range of 24 to 30 mm in boldface.

## DISCUSSION

The MIC and disk category agreement and error rates are based on proposed MIC and disk breakpoints, which have not yet been approved by FDA and/or CLSI. The overall category agreement rate of the 2-μg sulopenem disk compared to MIC for 2,856 *Enterobacterales* isolates was excellent (97.7%) and reflects minimal minor and major errors (0.9% and 0.04%, respectively). The 2-μg sulopenem disk provides for a wide separation of zone results based on distinctly resistant and susceptible isolates. The disk to MIC category agreement and error rates were based on analysis using an intermediate range ± 1 dilution, as described in CLSI M23 for challenge sets of organisms (M23). The consolidated MIC and disk data for *Enterobacterales* (*n* = 2,856) and E. coli (*n* = 2,148) incorporates a large set of clinical trial isolates, the majority of which are sulopenem susceptible. The analysis of the intermediate range ±1 dilution provides the necessary “buffer zone” based on inherent MIC and disk variation; however, the error rates are limited by relatively few isolates within this range. Additionally, the data presented are limited to a relatively small number of carbapenem-resistant isolates (including carbapenemase and noncarbapenemase carbapenem-resistant isolates); comparison of MIC and disk results for strains characterized by specific resistance markers is worth further investigation. The use of one manufacturer’s disk, which was based on availability and time line restrictions at the time of these studies, is another notable limitation. Further evaluation of the sulopenem disk included the establishment of QC ranges for antibiotic susceptibility testing. This is necessary to ensure accurate and reproducible reporting of results by laboratorians. The study described here met the CLSI M23 document requirements for tier 2 QC studies to establish MIC ranges for the sulopenem 2-μg disk (5). Ninety-nine percent of MIC values reported from the eight participating laboratories in this study were within the QC ranges shown in Table 2. The results from this multilaboratory QC study for sulopenem were approved by the CLSI Subcommittee on Antimicrobial Susceptibility and published in document M100Ed30 (1).

In summary, an accurate and reproducible sulopenem disk should be available to laboratories for disk diffusion testing of *Enterobacterales* isolates.
