# Supplementary material for: Sulopenem Disk Development, Quality Control Range, and MIC to Disk Result Correlation for Enterobacterales Isolates
Source: J Clin Microbiol. 2023 Jun 26;61(7):e00246-23. doi: 10.1128/jcm.00246-23 (PMC10358168; doi:10.1128/jcm.00246-23)
Supplement: Supplemental file 1 — Supplemental material. Download jcm.00246-23-s0001.docx, DOCX file, 0.04 MB [file jcm.00246-23-s0001.docx]

**Table S1. Sulopenem 5, 10, 20 µg Disk Development (CMI): Line Listing of MIC and Disk Results**

| **No** | **CMI ID** | **Organism** | **Phenotype/**  **Genotype** | **SUL**  **MIC**  **(mg/L)** | **SUL**  **20 µg**  **(mm)** | **SUL**  **10 µg**  **(mm)** | **SUL**  **5 µg**  **(mm)** |
| --- | --- | --- | --- | --- | --- | --- | --- |
| 1 | N10619 | *E. aerogenes* |  | 0.12 | 31 | 28 | 26 |
| 2 | N10620 | *E. aerogenes* |  | 0.12 | 32 | 29 | 27 |
| 3 | N10857 | *E. aerogenes* |  | 0.25 | 30 | 27 | 24 |
| 4 | N11476 | *E. aerogenes* |  | 0.12 | 30 | 27 | 25 |
| 5 | N11477 | *E. aerogenes* | AmpC | 16 | 12 | 8 | 6 |
| 6 | N11478 | *E. aerogenes* | AmpC | 16 | 17 | 10 | 6 |
| 7 | N10945 | *E. cloacae* |  | 0.03 | 31 | 29 | 27 |
| 8 | N10946 | *E. cloacae* |  | 0.06 | 31 | 28 | 25 |
| 9 | N10947 | *E. cloacae* | ESBL | 0.12 | 35 | 32 | 30 |
| 10 | N10930 | *E. cloacae* | ESBL | 0.12 | 33 | 30 | 28 |
| 11 | N10933 | *E. cloacae* | ESBL | 0.25 | 32 | 28 | 26 |
| 12 | N10935 | *E. cloacae* | ESBL | 0.25 | 31 | 28 | 25 |
| 13 | N10606 | *E. coli* |  | 1 | 29 | 27 | 24 |
| 14 | N10607 | *E. coli* |  | 0.03 | 34 | 32 | 30 |
| 15 | N10608 | *E. coli* |  | 0.03 | 37 | 34 | 31 |
| 16 | N10609 | *E. coli* |  | 1 | 30 | 26 | 23 |
| 17 | N10610 | *E. coli* |  | 0.015 | 38 | 35 | 32 |
| 18 | N10611 | *E. coli* |  | 0.03 | 36 | 33 | 30 |
| 19 | N10612 | *E. coli* |  | 0.03 | 37 | 33 | 31 |
| 20 | N10613 | *E. coli* |  | 1 | 23 | 20 | 18 |
| 21 | N10614 | *E. coli* |  | 1 | 24 | 20 | 18 |
| 22 | N10615 | *E. coli* |  | 0.03 | 35 | 32 | 30 |
| 23 | N10616 | *E. coli* |  | 0.03 | 35 | 30 | 28 |
| 24 | N10617 | *E. coli* |  | 0.03 | 35 | 33 | 30 |
| 25 | N10618 | *E. coli* |  | 0.03 | 31 | 28 | 25 |
| 26 | N10849 | *E. coli* |  | 0.015 | 33 | 31 | 29 |
| 27 | N10858 | *E. coli* |  | 0.06 | 38 | 32 | 28 |
| 28 | N11311 | *E. coli* | ESBL | 0.06 | 33 | 31 | 28 |
| 29 | N11312 | *E. coli* | ESBL | 0.03 | 37 | 34 | 31 |
| 30 | N11318 | *E. coli* | ESBL | 0.06 | 33 | 30 | 27 |
| 31 | N11321 | *E. coli* | ESBL | 0.03 | 34 | 32 | 29 |
| 32 | N11322 | *E. coli* | ESBL | 0.015 | 30 | 27 | 25 |
| 33 | N11323 | *E. coli* | ESBL | 0.06 | 32 | 29 | 27 |
| 34 | N11325 | *E. coli* | ESBL | 0.03 | 32 | 29 | 26 |
| 35 | N11327 | *E. coli* | ESBL | 0.06 | 33 | 31 | 29 |
| 36 | N11329 | *E. coli* | ESBL | 0.03 | 35 | 32 | 30 |
| 37 | N11331 | *E. coli* | ESBL | 0.03 | 33 | 30 | 29 |
| 38 | N11335 | *E. coli* | ESBL | 0.25 | 32 | 28 | 25 |
| 39 | N10390 | *K. pneumoniae* |  | 0.03 | 33 | 31 | 28 |
| 40 | N10391 | *K. pneumoniae* |  | 0.06 | 35 | 32 | 30 |
| 41 | N10392 | *K. pneumoniae* |  | 0.25 | 34 | 30 | 28 |
| 42 | N10393 | *K. pneumoniae* |  | 0.06 | 34 | 31 | 29 |
| 43 | N10637 | *K. pneumoniae* |  | 0.03 | 34 | 31 | 29 |
| 44 | N10638 | *K. pneumoniae* |  | 0.03 | 33 | 30 | 28 |
| 45 | N10639 | *K. pneumoniae* |  | 0.06 | 33 | 30 | 28 |
| 46 | N10640 | *K. pneumoniae* |  | 0.06 | 33 | 30 | 28 |
| 47 | N10641 | *K. pneumoniae* |  | 0.03 | 33 | 30 | 28 |
| 48 | N10642 | *K. pneumoniae* |  | 0.12 | 34 | 32 | 30 |
| 49 | N10837 | *K. pneumoniae* |  | 0.06 | 33 | 31 | 28 |
| 50 | N10839 | *K. pneumoniae* |  | 0.03 | 34 | 30 | 28 |
| 51 | N10840 | *K. pneumoniae* |  | 0.03 | 33 | 29 | 26 |
| 52 | N10842 | *K. pneumoniae* |  | 0.03 | 33 | 30 | 28 |
| 53 | N10843 | *K. pneumoniae* |  | 0.06 | 34 | 30 | 28 |
| 54 | N11375 | *K. pneumoniae* | ESBL | 0.06 | 33 | 30 | 27 |
| 55 | N11376 | *K. pneumoniae* | ESBL | 0.06 | 33 | 30 | 28 |
| 56 | N11377 | *K. pneumoniae* | ESBL | 0.12 | 31 | 28 | 26 |
| 57 | N11378 | *K. pneumoniae* | ESBL | 0.03 | 33 | 30 | 27 |
| 58 | N11379 | *K. pneumoniae* | ESBL | 0.03 | 34 | 31 | 29 |
| 59 | N11380 | *K. pneumoniae* | ESBL | 0.06 | 33 | 31 | 29 |
| 60 | N11381 | *K. pneumoniae* | ESBL | 0.03 | 32 | 30 | 28 |
| 61 | N11382 | *K. pneumoniae* | ESBL | 0.03 | 33 | 30 | 28 |
| 62 | N11383 | *K. pneumoniae* | ESBL | 0.03 | 33 | 30 | 28 |
| 63 | N11384 | *K. pneumoniae* | ESBL | 0.06 | 29 | 28 | 27 |
| 64 | N11385 | *K. pneumoniae* | ESBL | 0.06 | 31 | 29 | 27 |
| 65 | N11386 | *K. pneumoniae* | ESBL | 0.03 | 31 | 28 | 26 |
| 66 | N10352 | *M. morganii* |  | 0.25 | 31 | 28 | 25 |
| 67 | N10626 | *M. morganii* |  | 1 | 30 | 27 | 24 |
| 68 | N10627 | *M. morganii* |  | 1 | 31 | 27 | 23 |
| 69 | N10841 | *M. morganii* |  | 1 | 29 | 26 | 22 |
| 70 | N10860 | *M. morganii* |  | 1 | 28 | 24 | 21 |
| 71 | N9268 | *M. morganii* |  | 1 | 28 | 25 | 22 |
| 72 | N9269 | *M. morganii* |  | 1 | 28 | 25 | 21 |
| 73 | N9270 | *M. morganii* |  | 1 | 29 | 25 | 22 |
| 74 | N9271 | *M. morganii* |  | 1 | 28 | 25 | 22 |
| 75 | N9272 | *M. morganii* |  | 1 | 28 | 25 | 22 |
| 76 | N9273 | *M. morganii* |  | 1 | 29 | 26 | 22 |
| 77 | N9274 | *M. morganii* |  | 1 | 29 | 26 | 23 |
| 78 | N10644 | *P. mirabilis* |  | 0.12 | 29 | 27 | 26 |
| 79 | N10645 | *P. mirabilis* |  | 0.25 | 31 | 28 | 24 |
| 80 | N10646 | *P. mirabilis* |  | 0.03 | 35 | 33 | 31 |
| 81 | N10647 | *P. mirabilis* |  | 0.25 | 32 | 30 | 26 |
| 82 | N10838 | *P. mirabilis* |  | 0.06 | 34 | 31 | 29 |
| 83 | N10855 | *P. mirabilis* |  | 0.5 | 33 | 30 | 28 |
| 84 | N11296 | *P. mirabilis* | ESBL | 0.25 | 32 | 29 | 27 |
| 85 | N8470 | *P. vulgaris* |  | 0.5 | 28 | 26 | 24 |
| 86 | N8657 | *P. vulgaris* |  | 0.5 | 30 | 28 | 25 |
| 87 | N8658 | *P. vulgaris* |  | 0.12 | 32 | 29 | 26 |
| 88 | N9687 | *P. vulgaris* |  | 0.5 | 30 | 26 | 24 |
| 89 | N9698 | *P. vulgaris* |  | 0.25 | 29 | 26 | 25 |
| 90 | N9832 | *P. vulgaris* |  | 0.5 | 29 | 26 | 24 |
| 91 | N9899 | *P. vulgaris* |  | 1 | 29 | 27 | 24 |
| 92 | N10853 | *Providencia* spp. |  | 0.12 | 34 | 31 | 28 |
| 93 | N10854 | *Providencia* spp*.* |  | 0.12 | 35 | 32 | 28 |
| 94 | N8508 | *Providencia* spp*.* |  | 0.12 | 31 | 28 | 25 |
| 95 | N9510 | *Providencia* spp. |  | 0.12 | 31 | 28 | 25 |
| 96 | N9511 | *Providencia* spp*.* |  | 0.12 | 31 | 28 | 25 |
| 97 | N9512 | *Providencia* spp*.* |  | 0.12 | 31 | 28 | 25 |
| 98 | N10394 | *S. marcescens* |  | 0.5 | 30 | 27 | 24 |
| 99 | N10628 | *S. marcescens* |  | 2 | 30 | 27 | 24 |
| 10 | N10629 | *S. marcescens* |  | 0.25 | 30 | 27 | 25 |
| 10 | N10630 | *S. marcescens* |  | 2 | 29 | 26 | 24 |
| 10 | N10631 | *S. marcescens* |  | 0.5 | 32 | 28 | 25 |
| 10 | N10632 | *S. marcescens* |  | 0.5 | 31 | 27 | 25 |
| 10 | N9394 | *Salmonella* spp*.* |  | 0.03 | 36 | 33 | 30 |
| 10 | N9404 | *Salmonella* spp*.* |  | 0.03 | 36 | 33 | 30 |
| 10 | N9417 | *Salmonella* spp*.* |  | 0.03 | 34 | 32 | 30 |
| 10 | N10593 | *Salmonella* spp*.* |  | 0.03 | 34 | 32 | 30 |
| 10 | N10594 | *Salmonella* spp*.* |  | 0.03 | 32 | 30 | 28 |
| 10 | N10595 | *Salmonella* spp*.* |  | 0.03 | 32 | 29 | 26 |
| 11 | N10685 | *Shigella* spp*.* |  | 0.03 | 38 | 34 | 30 |
| 11 | N10686 | *Shigella* spp. |  | 0.03 | 35 | 33 | 30 |
| 11 | N10687 | *Shigella* spp*.* |  | 0.03 | 36 | 31 | 28 |
| 11 | N10689 | *Shigella* spp. |  | 0.015 | 34 | 32 | 29 |
| 11 | N8153 | *Shigella* spp*.* |  | 0.03 | 31 | 29 | 27 |
| 11 | N10028 | *Shigella* spp. |  | 0.03 | 36 | 33 | 31 |
| 11 | N10029 | *Shigella* spp*.* |  | 0.06 | 39 | 36 | 33 |

**Table S2. Sulopenem 2 and 5-µg Disk Development (Liofilchem) Study: Sulopenem (SUL) MIC and 2 µg disk results**

| N | Liofilchem ID | Organism | Phenotype/  Genotype | SUL  MIC  (mg/L | SUL  2 μg DISK  (mm) |
| --- | --- | --- | --- | --- | --- |
| 1 | VA25 | *E. coli* | ESBL | 0.064 | 26 |
| 2 | VA26 | *E. coli* | ESBL | 0.032 | 27 |
| 3 | VA27 | *E. coli* | ESBL | 0.064 | 27 |
| 4 | VA28 | *E. coli* | ESBL | 0.064 | 27 |
| 5 | VA29 | *E. coli* | ESBL | 0.064 | 29 |
| 6 | VA30 | *E. coli* | ESBL | 0.064 | 27 |
| 7 | VA31 | *E. coli* | ESBL | 0.064 | 27 |
| 8 | VA32 | *E. coli* | ESBL | 0.064 | 26 |
| 9 | VA33 | *E. coli* | ESBL | 0.125 | 25 |
| 10 | VA37 | *E. coli* | - | ≤0.016 | 31 |
| 11 | VA38 | *E. coli* | - | 0.064 | 28 |
| 12 | VA39 | *E. coli* | - | 0.032 | 28 |
| 13 | VA40 | *E. coli* | - | >16 | 6 |
| 14 | VA55 | *E. coli* | ESBL | >16 | 6 |
| 15 | VA56 | *E. coli* | - | 0.032 | 27 |
| 16 | VA57 | *E. coli* | - | 0.064 | 24 |
| 17 | VA60 | *E. coli* | - | 0.032 | 27 |
| 18 | VA61 | *E. coli* | - | 0.032 | 26 |
| 19 | VA62 | *E. coli* | - | 0.032 | 27 |
| 20 | VA64 | *E. coli* | ESBL | 0.064 | 27 |
| 21 | VA65 | *E. coli* | ESBL | 0.064 | 27 |
| 22 | VA66 | *E. coli* | ESBL | 0.064 | 27 |
| 23 | VA67 | *E. coli* | ESBL | 0.032 | 30 |
| 24 | VA68 | *E. coli* | ESBL | 0.032 | 27 |
| 25 | VA69 | *E. coli* | ESBL | ≤0.016 | 28 |
| 26 | VE55 | *K. pneumoniae* | KPC2/3 | >16 | 6 |
| 27 | VE56 | *K. pneumoniae* | KPC2/3 | >16 | 6 |
| 28 | VG68 | *K. pneumoniae* | VIM | >16 | 6 |
| 29 | VG69 | *E. coli* | NDM | >16 | 6 |
| 30 | VG70 | *E. cloacae* | NDM | >16 | 6 |
| 31 | VG71 | *K. pneumoniae* | VIM | 8 | 6 |
| 32 | VG72 | *E. coli* | NDM | 16 | 6 |
| 33 | VG73 | *E. coli* | OXA-48 | 4 | 11 |
| 34 | VG74 | *K. pneumoniae* | OXA-162 | 2 | 14 |
| 35 | VG75 | *K. pneumoniae* | VIM | 4 | 10 |
| 36 | VG76 | *K. pneumoniae* | NDM | >16 | 6 |
| 37 | VG77 | *K. pneumoniae* | VIM-1, KPC-2, TEM1, SHV-11 | >16 | 6 |
| 38 | VG78 | *E. coli* | OXA-48 | >16 | 6 |
| 39 | VG79 | *E. coli* | OXA-49 | 16 | 6 |
| 40 | VG81 | *E. cloacae* | - | 0.25 | 19 |
| 41 | VG81 | *E. cloacae* | - | 0.125 | 22 |
| 42 | VG82 | *E. coli* | VIM-4, KPC-2, CMY-4, TEM-1, SHV-1 | 16 | 6 |
| 43 | VG83 | *E. coli* | KPC | >16 | 6 |

**Sulopenem 2-µg Disk Development (Clinical Trial):**

**Number of Isolates Tested by Bacterial Species**

| **Species** | **N** |
| --- | --- |
| *Citrobacter amalonaticus* | 1 |
| *Citrobacter braakii* | 10 |
| *Citrobacter farmeri* | 1 |
| *Citrobacter freundii* | 38 |
| *Citrobacter koseri* | 14 |
| *Enterobacter asburiae* | 2 |
| *Enterobacter cloacae* | 41 |
| *Enterobacter cloacae* complex | 34 |
| *Enterobacter*, non-speciated | 4 |
| *Escherichia coli* | 2,148 |
| *Escherichia vulneris* | 1 |
| *Hafnia alvei* | 4 |
| *Klebsiella aerogenes* | 21 |
| *Klebsiella oxytoca* | 37 |
| *Klebsiella pneumoniae* | 302 |
| *Klebsiella variicola* | 25 |
| *Kluyvera ascorbata* | 1 |
| *Leclercia adecarboxylata* | 2 |
| *Lelliottia amnigena* | 4 |
| *Morganella morganii* | 30 |
| *Pantoea septica* | 1 |
| *Proteus hauseri* | 5 |
| *Proteus mirabilis* | 89 |
| *Proteus penneri* | 1 |
| *Proteus vulgaris* | 5 |
| *Providencia rettgeri* | 4 |
| *Providencia stuartii* | 4 |
| *Raoultella ornithinolytica* | 6 |
| *Raoultella planticola* | 4 |
| *Salmonella,* non-speciated | 1 |
| *Serratia marcescens* | 14 |
| *Serratia liquefaciens* | 1 |
| *Serratia,*non-speciated | 1 |
| **TOTAL** | **2,856** |
